# Diagnostic performance and clinical limitations of sentinel lymph node biopsy in head and neck melanoma: a 20-year retrospective cohort study

**DOI:** 10.1007/s00432-026-06499-5

**Published:** 2026-05-30

**Authors:** Lukas Lükewille, Susanne Wiegand, Asita Fazel, Katharina C. Kähler, Guido Radecker, Markus Hoffmann, within the HANSEATIC Head and Neck Oncology Collaboration

**Affiliations:** 1 Department of Otorhinolaryngology, Head and Neck Surgery, University Medical Center Schleswig-Holstein, Arnold-Heller-Straße 3, 24105 Kiel, Germany; 2 Department of Dermatology, Venereology and Allergology, University Medical Center Schleswig-Holstein, Kiel, Germany

**Keywords:** Sentinel lymph node biopsy, Head and neck melanoma, Lymphatic drainage, False-negative rate, SPECT/CT, Breslow thickness

## Abstract

**Purpose:**

Sentinel lymph node biopsy (SLNB) is a standard procedure for nodal staging in cutaneous melanoma. However, its diagnostic performance in the head and neck region remains limited due to complex lymphatic drainage patterns. This study aimed to evaluate the long-term diagnostic accuracy of SLNB in head and neck melanoma and to analyze anatomical and clinical factors associated with false-negative results.

**Methods:**

This retrospective single-center cohort study included patients with cutaneous head and neck melanoma who underwent SLNB between 2002 and 2022. Patients were stratified into two cohorts (2002–2011 and 2012–2022). Detection rates, sensitivity, negative predictive value, and false-negative rates were calculated. Overall survival was analyzed using Kaplan–Meier estimates and compared by log-rank testing.

**Results:**

A total of 189 patients were included. Sentinel lymph node detection significantly improved from 78.2% in the earlier cohort to 98.0% in the later cohort (*p* < 0.0001). Across the combined cohort, SLNB sensitivity was 72.5%, with a false-negative rate of 27.5%. False-negative events predominantly occurred in anatomically complex drainage regions. Sentinel lymph node status was not significantly associated with overall survival (*p* = 0.627).

**Conclusions:**

Despite substantial improvements in detection rates, SLNB in head and neck melanoma demonstrates clinically relevant limitations in diagnostic sensitivity. The observed false-negative rate highlights a risk of understaging, which may affect eligibility for adjuvant systemic therapies. SLNB findings should therefore be interpreted in the context of anatomical risk patterns and integrated into individualized follow-up strategies. The lack of an association between sentinel lymph node status and overall survival may reflect the impact of modern adjuvant systemic therapies and the distinct biological behavior of head and neck melanoma. However, this finding should be interpreted with caution given the limited sample size. These findings underscore the importance of procedural expertise and risk-adapted surveillance in this anatomically complex region.

## Introduction

Cutaneous melanoma of the head and neck accounts for approximately 20–30% of all melanoma cases and is characterized by distinct biological and anatomical features. Compared with melanomas of the trunk and extremities, head and neck melanomas exhibit more complex lymphatic drainage patterns, higher rates of early nodal involvement, and an overall less favorable prognosis (Amin et al. 2017; Eggermont et al. 2014; Garbe et al. 2022) These characteristics contribute to challenges in regional staging and may adversely affect oncologic outcomes.

Sentinel lymph node biopsy (SLNB) is an established staging procedure in cutaneous melanoma and provides essential prognostic information (Garbe et al. 2024; Swetter et al. 2019; Morton et al. 1992). In addition to its prognostic value, SLNB plays a pivotal role in guiding therapeutic decision-making, as sentinel lymph node status determines eligibility for adjuvant systemic therapies in patients with resected melanoma (Morton et al. 2006, 2014; Faries et al. 2017). While SLNB demonstrates high diagnostic accuracy in most anatomical regions, its performance in the head and neck remains inferior to that reported for melanomas of the trunk and extremities (Leiter et al. 2016; Eggermont et al. 2018; Weber et al. 2017). This limitation is clinically relevant, as inaccurate nodal staging may lead to understaging and potentially affect subsequent treatment strategies.

The reduced accuracy of SLNB in the head and neck is multifactorial. Highly variable and frequently overlapping lymphatic drainage pathways complicate preoperative mapping and increase the risk of failing to identify true first-echelon lymph nodes (Chao et al. 2003; Rosa et al. 2011; Hanks et al. 2020). In addition, the close anatomical proximity of multiple drainage basins, radiotracer shine-through effects, and the technical complexity of surgical dissection in anatomically crowded regions further compromise reliable sentinel lymph node identification (Chao et al. 2003; Rosa et al. 2011; Hanks et al. 2020). As a consequence, false-negative SLNB results occur more frequently in head and neck melanoma than in other anatomical locations, with important implications for staging accuracy and patient management (Lachiewicz et al. 2008; Spoerl et al. 2021; Pasha et al. 2023).

Over the past two decades, technical advances, most notably the introduction of hybrid imaging modalities such as SPECT/CT, along with increasing procedural standardization, have aimed to improve sentinel lymph node detection in head and neck melanoma (Karim et al. 2008; Miller et al. 2011; Veenstra et al. 2011; Downey et al. 2024). Several studies have demonstrated improved localization accuracy with advanced imaging techniques; however, whether these developments have translated into sustained improvements in overall diagnostic performance remains incompletely defined (Karim et al. 2008; Miller et al. 2011; Veenstra et al. 2011; Downey et al. 2024). Moreover, the anatomical patterns and underlying mechanisms associated with false-negative SLNB findings remain insufficiently characterized, despite their critical relevance for risk stratification, treatment planning, and follow-up strategies.

The present study provides a comprehensive 20-year evaluation of SLNB in patients with head and neck melanoma treated at a single tertiary academic center. By comparing two consecutive cohorts from 2002–2011 and 2012–2022, we assess temporal trends in sentinel lymph node detection rates, lymphatic drainage patterns, nodal metastasis, false-negative mechanisms, and survival outcomes. The aim of this analysis is to define the diagnostic strengths and limitations of SLNB in this anatomically complex region and to identify clinically relevant factors associated with false-negative results.

## Materials and methods

A retrospective single-center cohort study was conducted at the Department of Otorhinolaryngology, Head and Neck Surgery, University Medical Center Schleswig–Holstein (UKSH), Campus Kiel. All patients with histologically confirmed cutaneous melanoma of the head and neck who underwent sentinel lymph node biopsy (SLNB) between January 2002 and December 2022 were identified from institutional clinical records.

To evaluate temporal changes in diagnostic performance, the study period was divided into two consecutive cohorts. The first cohort (2002–2011) represents an earlier institutional patient group that has been reported previously and was included as a historical reference. The second cohort (2012–2022) reflects a later institutional period characterized by standardized documentation, routine use of advanced imaging modalities, and complete staging information, and constituted the primary dataset for detailed analyses in the present study.

Inclusion criteria were the presence of a primary, non-metastatic cutaneous melanoma located within the anatomical boundaries of the head and neck region, performance of SLNB with localization of at least one sentinel lymph node within the head and neck region, and availability of valid informed consent for the use of clinical data within the institutional broad consent framework and the study was approved by the local ethics committee (D 505/22). Exclusion criteria included synchronous or metachronous multiple primary malignant melanomas and incomplete or missing documentation that precluded reliable evaluation of clinical course or nodal status.

Patients treated between 2012 and 2022 with complete staging and follow-up information formed the primary cohort for analyses of sentinel lymph node status, diagnostic performance parameters, and survival outcomes. Patients treated between 2002 and 2011 were included for comparative analyses of sentinel lymph node detection rates, lymphatic drainage patterns, and the occurrence of false-negative SLNB events.

### Sentinel lymph node detection and surgical technique

Preoperative lymphatic mapping was performed using peritumoral injections of technetium-99 m–labeled nanocolloid. Planar lymphoscintigraphy was used for sentinel lymph node localization throughout the entire study period. In the later study years, hybrid single-photon emission computed tomography combined with computed tomography (SPECT/CT) was increasingly implemented to improve anatomical localization and depth assessment of sentinel lymph nodes.

Intraoperative identification of sentinel lymph nodes was performed using a handheld gamma probe. All lymph nodes demonstrating radiotracer uptake of at least 10% of the maximum activity (“hottest node”) were excised. The number of harvested sentinel lymph nodes and their anatomical localization were documented according to the standard cervical neck level classification.

### Histopathological analysis

Excised sentinel lymph nodes were fixed in formalin and processed according to institutional histopathological protocols. Serial sectioning with hematoxylin–eosin staining was performed and supplemented by immunohistochemical analysis using S-100 protein, HMB-45, and Melan-A, as indicated.

Sentinel lymph node status was classified as true positive in the presence of histologically confirmed metastatic involvement. Cases were classified as true negative when no metastatic disease was detected in the sentinel lymph node and no subsequent regional nodal recurrence occurred during follow-up. A false-negative result was defined as the development of regional nodal metastasis following an initially negative sentinel lymph node biopsy. Non-sentinel lymph nodes removed during completion neck dissection were documented and analyzed separately.

### Clinical follow-up and outcome measures

Patients underwent regular follow-up examinations in accordance with contemporary guideline-based recommendations, including both dermatological and otorhinolaryngological surveillance. As part of routine clinical follow-up, patients received both dermatological and otorhinolaryngological examinations, with routine cervical ultrasound as an integral component of the ENT follow-up. Additional cross-sectional imaging was performed when clinically indicated. Information on recurrence patterns, including local, locoregional, and distant recurrence, as well as survival outcomes, was retrieved from electronic medical records.

The primary endpoints of the study were sentinel lymph node detection rate, diagnostic sensitivity, false-negative rate, negative predictive value, anatomical distribution of sentinel lymph node drainage patterns, and comparison of tumor characteristics between true-positive, true-negative, and false-negative groups. Secondary endpoints included findings at completion neck dissection and overall survival.

### Statistical analysis

Statistical analyses were performed using IBM SPSS Statistics. Categorical variables were analyzed using Fisher’s exact test or the chi-square test, as appropriate. Continuous variables were compared using the independent samples t-test or the Mann–Whitney U-test, depending on data distribution. Homogeneity of variances was assessed using Levene’s test, and Welch correction was applied when indicated.

Diagnostic performance parameters were calculated using standard definitions, including sensitivity (TP/[TP + FN]), false-negative rate (FN/[TP + FN]), and negative predictive value (TN/[TN + FN]). Receiver operating characteristic (ROC) analysis was performed to evaluate the discriminatory performance of Breslow thickness for sentinel lymph node positivity.

Survival outcomes were analyzed using Kaplan–Meier estimates. Differences between groups were assessed using the log-rank test, log-rank trend test, and Gehan–Breslow–Wilcoxon test, as appropriate. All statistical tests were two-sided, and a *p*-value of less than 0.05 was considered statistically significant.

## Results

### Patient characteristics

Between 2012 and 2022, a total of 102 patients underwent sentinel lymph node biopsy (SLNB) for cutaneous melanoma of the head and neck. In 100 patients, at least one sentinel lymph node (SLN) was successfully identified. For analyses dependent on SLN status, only cases with complete pathological evaluation were included, resulting in a final study cohort of 100 patients.

The mean age of the study cohort was 66 years (range 26–87). Primary tumors were most frequently located in the facial region (52.9%), followed by the ear (18.6%), the cervical/nuchal/submandibular region (14.7%), and the scalp (13.7%). Mean Breslow thickness was 2.41 mm.

For comparative analyses, a historical cohort of 87 patients treated between 2002 and 2011 at the same institution was included. In this cohort, sentinel lymph node detection was successful in 68 patients (78.2%). Comparison between cohorts demonstrated similar demographic characteristics and follow-up duration, indicating good baseline comparability.

### SLN detection and anatomical distribution

Sentinel lymph node detection improved significantly over time. Detection rates increased from 78.2% in the historical cohort (2002–2011) to 98.0% in the contemporary cohort (2012–2022) (Fisher’s exact test, *p* < 0.0001; odds ratio 13.97; 95% confidence interval 3.37–61.62).

In the contemporary cohort (n = 100), SLNs were most frequently identified in the parotid region (30.0%) and cervical level II (28.0%), followed by level I (19.0%), level V (16.0%), level III (4.0%), and level IV (3.0%). Sentinel lymph nodes located in more than one anatomical level were detected in 33.3% of patients.

**Fig. 1:**
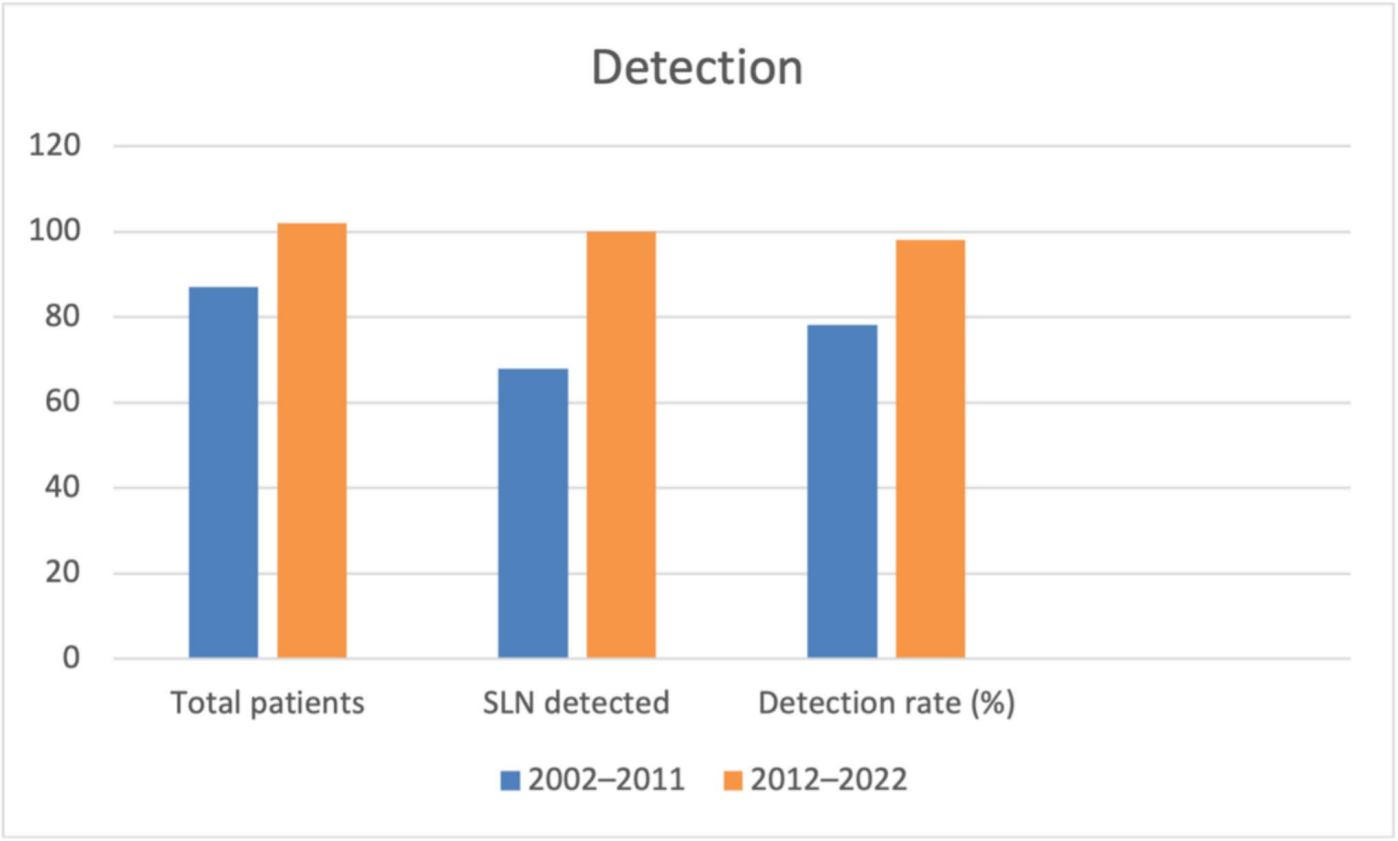
Sentinel lymph node detection by study period. Total number of patients, number of patients with successful sentinel lymph node (SLN) detection, and corresponding detection rates are shown for the 2002–2011 and 2012–2022 cohorts. SLN detection was significantly higher in the contemporary cohort

Comparison with the historical cohort revealed a significant shift in lymphatic drainage patterns (Fisher’s exact test, *p* = 0.0458), characterized by an increased proportion of auricular and parotid drainage pathways and a relative decline in cervical, nuchal, and submandibular drainage regions (Fig. 1).

### Histopathologic findings of SLNs

Among the 100 evaluable patients treated between 2012 and 2022, 81 patients (81%) had histologically negative sentinel lymph nodes (true negative). Eleven patients (11%), seven patients (7%), and one patient (1%) had one, two, and four positive sentinel lymph nodes, respectively. No cases with three positive sentinel lymph nodes were observed.

Comparison with the historical cohort (2002–2011) showed no significant difference in the distribution of sentinel lymph node positivity when categorized as 0, 1, or ≥ 2 positive nodes (Chi-square test, χ^2^(2) = 1.95; *p* = 0.3773).

### False-negative (FN) SLNB findings

Across the combined 20-year dataset (2002–2022), 11 false-negative (FN) SLNB cases were identified, defined as the development of regional nodal metastasis following an initially negative SLNB. Eight false-negative cases occurred in the 2012–2022 cohort and three in the historical cohort.

In false-negative cases, subsequent regional nodal metastases showed a distinct anatomical distribution. Cervical level II was the most frequently involved nodal basin (63.6%), followed by level V (18.2%), level I (9.1%), and level III (9.1%). No false-negative cases involved the parotid basin or deep cervical or nuchal nodal regions.

Primary tumors associated with false-negative SLNB were predominantly located in the facial region (81.8%).

Comparative analyses demonstrated significant differences in nodal localization patterns between groups. In true-positive cases, nodal metastases were more frequently observed in deeper drainage pathways, predominantly cervical level V and cervical/nuchal regions, whereas in true-negative cases the initially identified sentinel lymph nodes were most commonly located in the parotid basin and cervical level II (Fisher’s exact test, *p* < 0.0001). False-negative cases showed pronounced clustering of subsequent nodal metastases in cervical level II compared with both true-positive cases (*p* < 0.0001) and true-negative cases (*p* = 0.036).

### Tumor characteristics and SLN positivity

Mean Breslow thickness differed significantly between true-positive and true-negative cases. True-positive tumors demonstrated a mean Breslow thickness of 3.23 mm (95% confidence interval 2.37–4.10), whereas true-negative tumors showed a mean thickness of 2.31 mm (95% confidence interval 2.01–2.61) (t(154) = 2.46; *p* = 0.015; η^2^ = 0.038).

Receiver operating characteristic analysis confirmed strong discriminative performance of Breslow thickness for sentinel lymph node positivity (area under the curve 0.9815; 95% confidence interval 0.9711–0.9919; *p* < 0.0001).

**Fig. 2:**
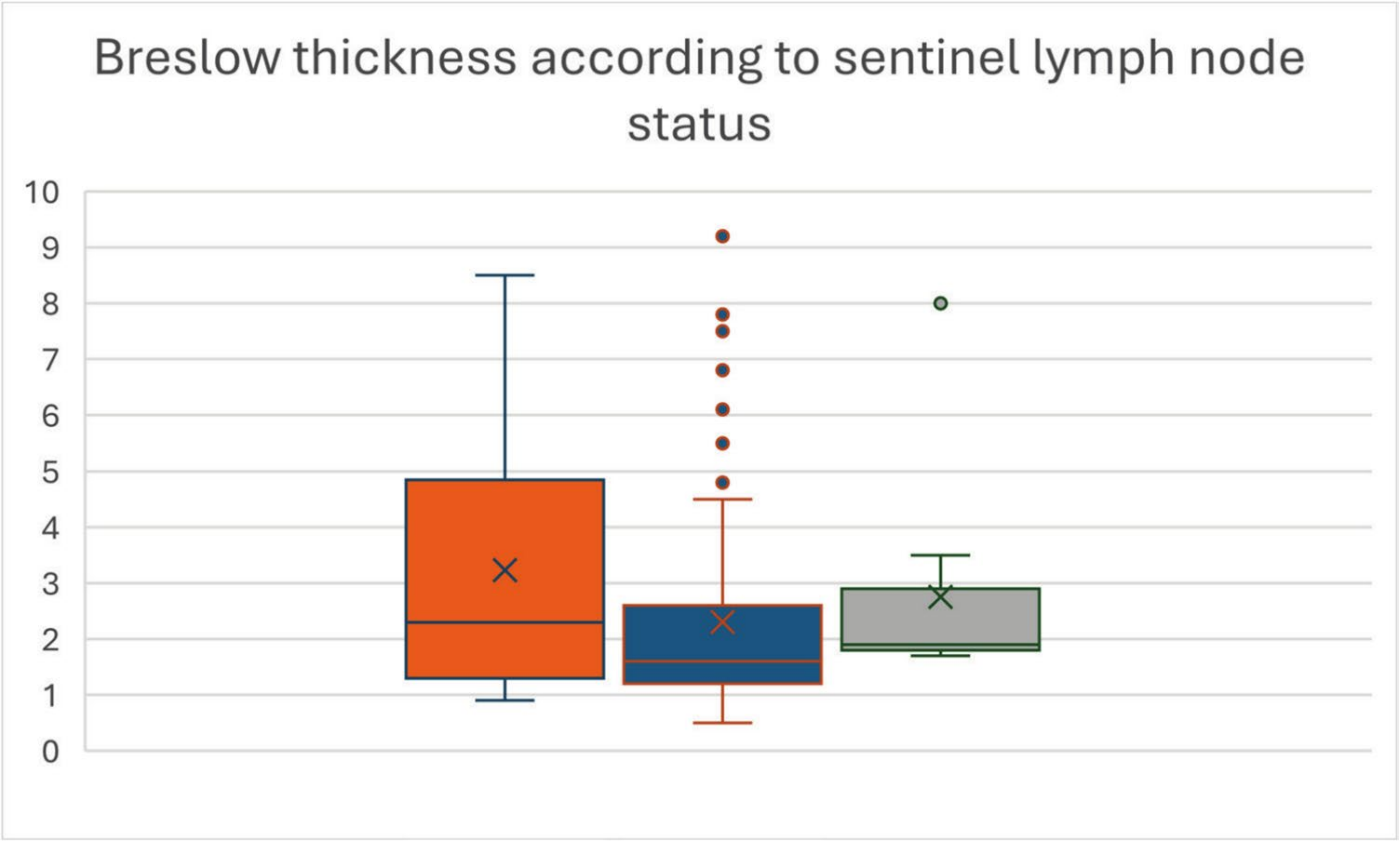
Breslow thickness (mm) by sentinel lymph node status. Shown are the distributions of Breslow thickness in true-positive (orange), true-negative (blue), and false-negative (gray) cases

No significant differences in Breslow thickness were observed between true-positive and false-negative cases (*p* = 0.536) or between false-negative and true-negative cases (*p* = 0.411) (Fig. 2).

### Neck dissection findings

In the 2012–2022 cohort, 17 of 102 patients (16.67%) underwent completion neck dissection. Among these patients, 14 (82.4%) showed no additional metastatic lymph nodes, whereas three patients demonstrated further nodal involvement, with one, two, and eight additional positive lymph nodes, respectively.

Of the 18 patients with sentinel lymph node–positive disease during this period, 15 underwent completion neck dissection.

### Metastatic disease and survival outcomes

During follow-up, 32 patients (32.7%) developed metastatic disease, including five cases (16%) of local recurrence, 14 cases (44%) of locoregional metastases, and 23 cases (72%) of distant metastases. Combined metastatic patterns were observed in 31% of patients.

Primary tumors in patients who developed metastatic disease were most frequently located in the facial region (47%) and the scalp (25%).

**Fig. 3:**
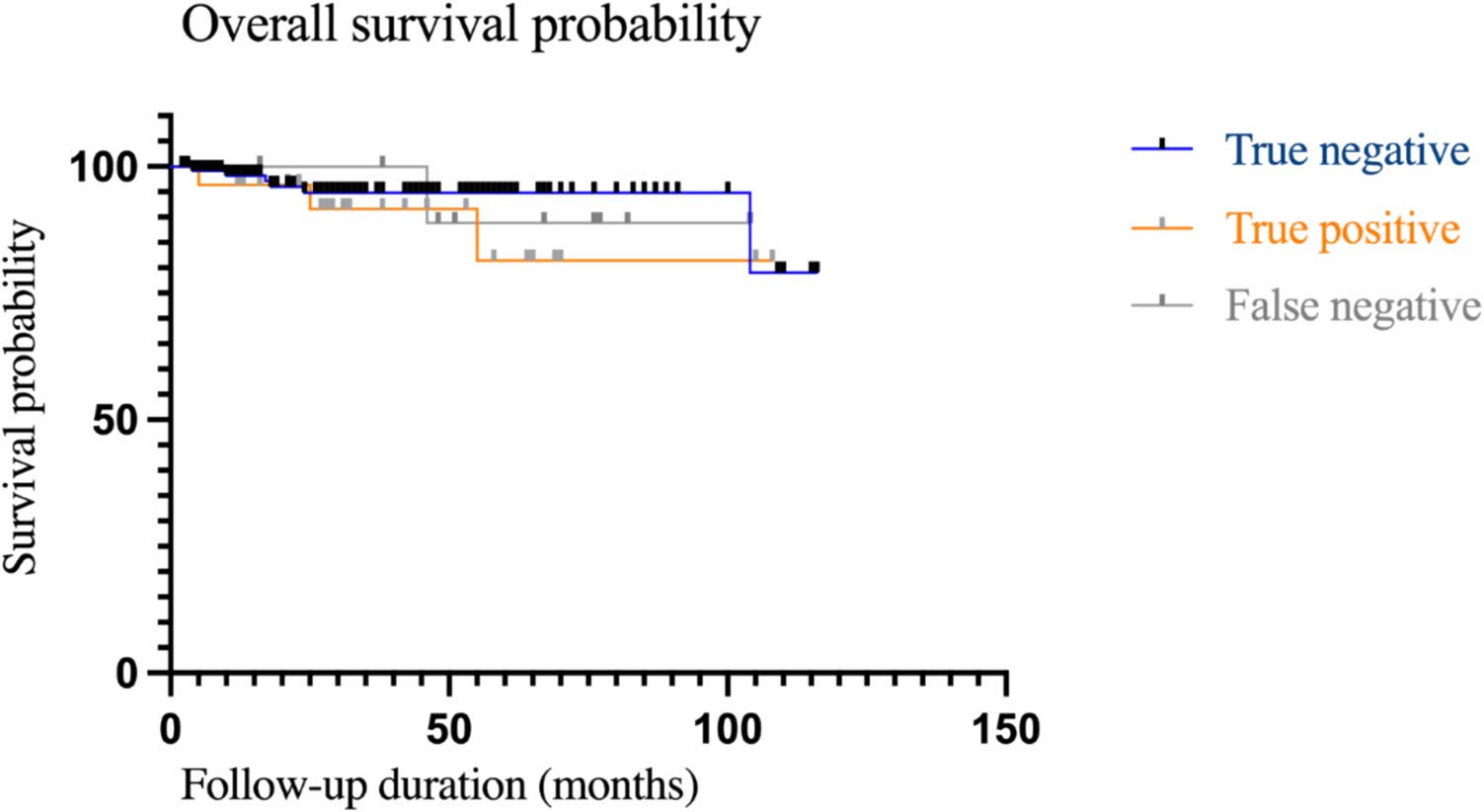
Kaplan–Meier analysis of overall survival by sentinel lymph node status. Kaplan–Meier curves illustrate estimated overall survival over the follow-up period stratified by sentinel lymph node status (true negative, true positive, and false negative). No significant difference in overall survival was observed between the three groups (log-rank test, *p* = 0.627)

Kaplan–Meier survival analysis demonstrated no significant association between sentinel lymph node status and overall survival (log-rank test *p* = 0.627; log-rank trend test *p* = 0.507; Gehan–Breslow–Wilcoxon test *p* = 0.670). No significant differences in overall survival were observed between true-positive, true-negative, and false-negative groups (Fig. 3).

## Discussion

This 20-year evaluation of sentinel lymph node biopsy (SLNB) in cutaneous melanoma of the head and neck demonstrates that, despite substantial advances in imaging and procedural standardization, the diagnostic performance of SLNB in this anatomically complex region remains limited. Most notably, while the introduction of hybrid SPECT/CT imaging was associated with a marked increase in sentinel lymph node (SLN) detection rates, sensitivity and false-negative (FN) rates did not show a comparable improvement. These findings highlight that improved detection does not necessarily translate into improved diagnostic accuracy and underscore the multifactorial determinants of SLNB performance in the head and neck (Karim et al. 2008; Miller et al. 2011; Veenstra et al. 2011; Downey et al. 2024).

A key finding of this study is the significant increase in SLN detection rates in the contemporary cohort (98.0%). Although advances in preoperative imaging contributed to this improvement, detection rates cannot be attributed to imaging alone. Procedural consistency and surgical expertise appear to play a critical role. Approximately 90% of SLNB procedures in the later cohort were performed by a single experienced surgeon, whereas the earlier cohort involved multiple surgeons with varying levels of experience. SLNB in the head and neck is highly operator-dependent, requiring precise interpretation of lymphoscintigraphic findings and meticulous intraoperative exploration. Previous studies have demonstrated that surgeon experience significantly improves SLN detection and reduces variability, particularly in anatomically challenging regions such as the parotid basin and cervical level II (Chao et al. 2003; Rosa et al. 2011; Hanks et al. 2020). The near-complete detection rate observed in the contemporary cohort therefore likely reflects the combined effect of improved imaging and a pronounced experienced-surgeon effect. The absence of a corresponding improvement in sensitivity supports the interpretation that detection alone is not sufficient to overcome the intrinsic anatomical complexity of lymphatic drainage in this region.

The anatomical distribution of SLNs observed in this study provides further insight into the mechanisms underlying FN outcomes. False-negative cases showed a clear clustering in cervical level II, whereas true-positive cases were more frequently associated with deeper drainage pathways, particularly cervical level V and nuchal regions. Cervical level II represents a region characterized by highly variable and overlapping lymphatic drainage, as well as proximity to the primary injection site, which may result in radiotracer shine-through and hinder accurate localization (Chao et al. 2003; Rosa et al. 2011; Hanks et al. 2020). The predominance of facial primaries among FN cases further supports the relevance of this anatomical vulnerability. In contrast, the absence of FN events in the parotid basin suggests that, despite technical challenges, parotid-directed drainage pathways may be more consistently identified when adequately explored. These findings emphasize that anatomical factors, rather than tumor biology alone, play a central role in FN SLNB outcomes.

In routine clinical practice, lymphatic mapping is frequently performed after prior excision of the primary tumor, which may alter local lymphatic pathways. While this effect may be negligible in melanomas of the trunk and extremities, even subtle changes in tissue architecture or injection geometry may have greater impact in the anatomically dense head and neck region. In particular, facial melanomas are often initially excised outside specialized centers, potentially introducing additional variability in lymphatic mapping. Although this factor cannot be quantified in the present study, it represents a plausible contributor to the increased FN rates observed and warrants further investigation.

Tumor-related factors also influenced SLNB performance. Consistent with previous reports, Breslow thickness was significantly associated with SLN positivity (Lachiewicz et al. 2008; Spoerl et al. 2021; Pasha et al. 2023). Importantly, no significant differences in tumor thickness were observed between true-positive and FN cases, indicating that FN results are not primarily driven by tumor biology but rather by anatomical and technical factors. In addition, the higher proportion of metastatic disease observed in scalp and cervical or nuchal melanomas is in line with prior studies describing more aggressive behavior and distinct biological characteristics in these subsites (Amin et al. 2017; Eggermont et al. 2014; Garbe et al. 2022).

Beyond its prognostic role, SLNB has direct implications for therapeutic decision-making. Sentinel lymph node status determines eligibility for adjuvant systemic therapies, including immune checkpoint inhibitors and targeted therapies, which are recommended from specific AJCC stages onward. Recent 2024 European consensus guidelines further emphasize the role of SLNB as an essential prerequisite for accurate stage classification and treatment decision-making, particularly regarding eligibility for adjuvant systemic therapies (Garbe et al. 2024). In this context, false-negative SLNB results may lead to clinically relevant understaging, potentially delaying or precluding the initiation of effective systemic treatment. The diagnostic limitations observed in this study therefore have direct consequences for patient management and underscore the importance of careful interpretation of negative SLNB findings, particularly in high-risk anatomical regions.

In the present analysis, SLN status was not associated with overall survival. This finding should be interpreted with caution given the limited sample size, the small number of false-negative events, and the limited ability to control for the confounding effects of modern adjuvant and palliative systemic therapies. In addition, it likely reflects the evolving treatment landscape, as modern adjuvant and palliative systemic therapies, particularly immune checkpoint inhibitors, have been shown to significantly improve survival outcomes in melanoma (Morton et al. 2006, 2014; Faries et al. 2017). These advances may attenuate the prognostic impact of nodal status observed in earlier treatment eras. Furthermore, head and neck melanoma is known to exhibit a higher propensity for early hematogenous dissemination compared with melanomas of other anatomical sites, which may further reduce the relative prognostic significance of regional nodal involvement.^1^–.^3^

The interpretation of neck dissection findings must also consider the paradigm shift in surgical management following the results of MSLT-II and DeCOG-SLT (Garbe et al. 2024; Swetter et al. 2019; Morton et al. 1992). Whereas completion lymph node dissection was previously performed routinely in SLN-positive patients, contemporary management favors a risk-adapted approach incorporating tumor burden, number of positive nodes, extracapsular extension, and the feasibility of structured ultrasound surveillance. Accordingly, the low rate of additional nodal metastases observed in this study likely reflects current treatment strategies rather than limitations of SLNB itself.

Taken together, these findings have several important clinical implications. First, SLNB remains a valuable staging procedure in head and neck melanoma, but its diagnostic limitations must be explicitly acknowledged, particularly for tumors draining to cervical level II. Second, hybrid SPECT/CT imaging and procedural standardization substantially improve SLN detection and should be considered essential components of contemporary SLNB practice in this region. Third, the risk of false-negative results remains clinically relevant, necessitating risk-adapted postoperative surveillance strategies, especially in patients with facial primaries and negative SLNB findings. Finally, surgical expertise and procedural consistency represent key determinants of diagnostic quality, supporting the centralization of SLNB for head and neck melanoma in specialized, high-volume centers.

### Limitations

This study has several limitations inherent to its retrospective design. First, although the overall cohort size was substantial, subgroup analyses, particularly for false-negative (FN) cases, were limited by small sample sizes, reducing statistical power and limiting the ability to perform robust multivariate analyses or detect subtle effects. Second, the 20-year study period encompasses significant technological, procedural, and guideline-related evolution, most notably the introduction of SPECT/CT imaging, evolving AJCC staging classifications, and changing indications for sentinel lymph node biopsy. Although the analysis was stratified into two temporal cohorts, residual heterogeneity in diagnostic pathways cannot be fully excluded. These developments may have influenced patient selection and referral patterns over time, introducing potential selection bias that cannot be fully controlled in a retrospective cohort. As a tertiary referral center serving a large geographic region in Schleswig–Holstein, Northern Germany, many patients were referred from distant areas, and reliable assessment of nodal recurrence required regular follow-up examinations at our institution. Consequently, only patients with sufficiently documented institutional follow-up could be included in analyses requiring reliable evaluation of false-negative events, which may have contributed to selection bias. Third, the identification of false-negative cases relied on clinical and radiologic follow-up rather than routine histopathological verification, which may underestimate microscopic nodal disease present at the time of SLNB. However, regular dermatological and otorhinolaryngological follow-up, including routine cervical ultrasound, likely improved the detection of non-palpable regional nodal recurrence and strengthened the validity of false-negative rate calculations. Fourth, molecular ultrastaging using techniques such as RT-PCR was not routinely performed. Although molecular analysis may detect occult micrometastatic disease in histologically negative sentinel lymph nodes, this approach is not part of standard clinical practice due to limited standardization and uncertain therapeutic relevance. Therefore, histopathological assessment with conventional histology and immunohistochemistry reflects current real-world diagnostic standards, although a slight underestimation of true diagnostic sensitivity cannot be excluded. Fifth, completion neck dissection was not uniformly performed in all SLN-positive patients in the earlier cohort, potentially limiting assessment of the true extent of nodal tumor burden. In addition, detailed quantification of sentinel node tumor burden was not uniformly available across the study period, reflecting evolving pathological reporting standards. This limits a more granular analysis of risk-adapted decision-making for completion neck dissection, particularly in the context of changing therapeutic algorithms. Sixth, as a single-center study, the results reflect local surgical expertise and institutional protocols and may not be fully generalizable to other practice settings.

Finally, the inherent anatomical complexity of the head and neck region introduces unavoidable sources of measurement bias. Minor variations in injection technique, imaging interpretation, or intraoperative exposure may influence SLN detection in ways that are difficult to capture in retrospective datasets. Prospective, multicenter studies are therefore warranted to validate the anatomical drainage patterns and false-negative mechanisms identified in the present analysis.

## Conclusion

Sentinel lymph node biopsy (SLNB) remains an important staging tool in head and neck melanoma. While advances in imaging and procedural standardization have significantly improved sentinel lymph node detection, diagnostic sensitivity remains limited due to anatomical complexity. False-negative results occur predominantly in specific drainage regions, particularly cervical level II, and may lead to clinically relevant understaging with potential implications for treatment decision-making.

Tumor thickness remains a strong predictor of sentinel node positivity but does not distinguish between true-positive and false-negative findings, underscoring the role of anatomical and technical factors. The lack of an association between sentinel lymph node status and overall survival should be interpreted with caution and may reflect both the limited sample size and the impact of modern systemic therapies.

These findings highlight the need for careful interpretation of negative SLNB results and support risk-adapted follow-up strategies, particularly in anatomically high-risk regions.

## Data Availability

The data supporting the findings of this study are available within the article. The anonymized data generated and analyzed during the current study are available upon reasonable request to qualified researchers.

## Abbreviations

AJCC: American joint committee on cancer
CT: Computed tomography
FN: False negative
FNR: False negative rate
HMB-45: Human melanoma black 45
KM: Kaplan–Meier
LN: Lymph node
MSLT: Multicenter selective lymphadenectomy trial
ND: Neck dissection
NPV: Negative predictive value
OS: Overall survival
RP / TP: True positive
RN / TN: True negative
S-100: S-100 protein
SLN: Sentinel lymph node
SLNB: Sentinel lymph node biopsy
SPECT/CT: Single-photon emission computed tomography/computed tomography
SPSS: Statistical package for the social sciences
